# Meetings of Societies

**Published:** 1919

**Authors:** 


### Meetings of Societies.

*Annual Meeting, December 11th,* 1918.

After a vote of thanks to the retiring President, Mr. F. Lace, had been passed, Major R. G. Poole Lansdown took the chair, and gave an introductory address (*see* p. 33). Later a cordial vote of thanks was given to Major Lansdown for the very interesting and instructive address he had delivered.

The following are the officers of the Society for the Session 1918–19: President—R. G. P. Lansdown, M.D., B.S. Durh.; President-Elect—I. Walker Hall, M.D. Vict. Committee (*ex officio*)—Frederick Lace (retiring President); P. Watson-Williams, M.D. Lond. (Editor of *Journal*); C. K. Rudge (Hon. Librarian); J. M. Fortescue-Brickdale, M.A., M.D., B.Ch. Oxon. (Editorial Secretary). (*Elected*)—George Parker, M.A., M.D. Cantab.; R. Shingleton Smith, M.D., B.Sc. Lond. (*ex Presidents*); J. Lacy Firth, M.D., M.S. Lond.; J. O. Symes, M.D. Lond.; L. E. V. Every-Clayton, M.D., B.S. Lond.; W. A. Smith, M.A., M.B.; T. Carwardine, M.S.; D. C. Rayner. Hon. Secretary—A. J. Wright, M.B., B.S. Lond.

*April 9th,* 1919.

The President, MAJOR R. G. POOLE LANSDOWN, in the Chair.

The meeting was devoted to a discussion of **The principles and practice of postgraduate study and instruction in the University and Medical Institutions of Bristol.**

